# Ischemic Stroke as the Initial Presentation of an Underlying Neuroinfectious Disease

**DOI:** 10.7759/cureus.118974

**Published:** 2026-11-02

**Authors:** Gurpreet Sehmbi, Meerat Abbas, Mohmedshahid Patel, Ahamad Hassan, Rabee Jamali

**Affiliations:** 1 Department of Neurology, Leeds Teaching Hospitals NHS Trust, Leeds, GBR

**Keywords:** cerebral vasculitis, hsv-2, ischemic stroke, magnetic resonance contrast-enhanced vessel wall imaging, multifocal infarctions, neurosyphilis

## Abstract

Neuroinfectious cerebral vasculitis is a rare but potentially underrecognized cause of ischemic stroke. Its clinical and neuroimaging features may closely mimic those of common cerebrovascular disorders, posing a diagnostic challenge. We describe two patients who presented with ischemic stroke, one with an atypical presentation and the other with recurrent episodes, in whom advanced neuroimaging and cerebrospinal fluid (CSF) analysis were pivotal in establishing the diagnosis.

A 55-year-old man presented with a two-month history of nonfocal symptoms and was found to have a right posterior cerebral artery infarct with multifocal intracranial stenoses. Syphilis serology was positive in both blood and CSF. High-resolution magnetic resonance vessel wall imaging (HR-VWI) demonstrated multifocal vessel irregularities suggestive of inflammatory vasculopathy, although the initial clinical suspicion was ischemic stroke.

A 48-year-old woman presented with recurrent posterior circulation strokes and progressively worsening neurological symptoms. HR-VWI again revealed vessel irregularities, prompting further investigation with CSF analysis, which identified herpes simplex virus 2 (HSV-2).

These cases highlight the importance of considering neuroinfectious etiologies in patients presenting with unexplained, multifocal, or recurrent ischemic strokes. Advanced neuroimaging techniques, such as HR-VWI, may help identify intracranial vasculitic changes and prompt further targeted investigations, including CSF analysis, which is essential for establishing a neuroinfectious diagnosis. Given the potentially treatable nature of these conditions, early recognition and initiation of pathogen-specific therapy, supported by a multidisciplinary approach, are essential to optimizing clinical outcomes.

## Introduction

Neuroinfectious cerebral vasculitis is a rare but recognized cause of stroke, occurring more frequently in younger individuals and those with atypical presentations. Although the pathogenesis varies among causative pathogens, neuroinfectious conditions are important differential diagnoses to consider in patients with atypical or unexplained stroke presentations, as early recognition can facilitate targeted treatment and may reduce the risk of significant long-term disability. The underlying pathophysiology varies depending on the pathogen but often involves vascular inflammation, leading to endothelial injury, arterial stenosis, and cerebral infarction. These conditions may mimic more common vascular or autoimmune disorders. Differentiation may be facilitated by advanced radiological techniques, such as magnetic resonance imaging (MRI) and high-resolution vessel wall imaging (HR-VWI), which can help distinguish inflammatory vasculopathy from atherosclerotic disease by identifying differences in vessel wall thickening patterns. Despite advances in diagnostic modalities and increasing clinical awareness of neuroinfectious conditions, their role as potential causes of ischemic stroke remains insufficiently recognized in clinical practice [1,2].

We present two patients who were admitted to the hospital with atypical stroke syndromes and were subsequently diagnosed with cerebral vasculitis secondary to *Treponema pallidum* (neurosyphilis) and herpes simplex virus 2 (HSV-2), respectively. In both cases, advanced MR techniques contributed to the diagnostic evaluation.

## Case presentation

Case 1

A 55-year-old man presented with a two-month history of diplopia, headaches, and ataxia. His medical history included alcohol dependence, previous pancreatitis, and depression. Initial examination revealed left-sided hemianopia, nystagmus on left lateral gaze, impaired coordination on the left side, and 4+/5 strength in the left upper limb. Coordination was preserved on the right side.

Non-contrast computed tomography (CT) of the head confirmed a right posterior cerebral artery (PCA) infarct. Laboratory investigations revealed elevated cholesterol levels and prediabetes. Further investigations, including CT angiography, demonstrated multifocal intracranial arterial stenoses, which were initially attributed to atherosclerosis. Additional investigations, including echocardiography, ambulatory electrocardiography (ECG), blood-borne virus screening (HIV and hepatitis B and C), an autoimmune panel (including anti-Ro60, anti-Ro52, anti-La, anti-Sm, anti-Sm/RNP, anti-RNP68, anti-Scl-70, anti-Jo-1, anti-centromere, anti-chromatin, anti-ribosomal P, anti-myeloperoxidase, and anti-proteinase 3 antibodies), and total homocysteine measurement, were unremarkable (Table 1).  

**Table 1:** Summary of laboratory findings in Case 1, with corresponding reference ranges

Investigation	Result	Units	Reference range
Plasma viscosity	1.66	mPa/S	1.50-1.72
Total cholesterol	6.68	mmol/L	<2.3
Hemoglobin A1c	46	mmol/mol	0-41
Total homocysteine	18	umol/L	0-18
Thyroid-stimulating hormone	2.12	mIU/L	0.2-4.3
Rheumatoid factor	<8	IU/mL	0-14
Autoimmune screen			
Anti-nuclear antibody screen	Negative		Negative
Anti-neutrophil cytoplasmic antibody (ANCA) myeloperoxidase	<0.20	AI	<0.99
ANCA proteinase 3	<0.20	AI	<0.99
Human immunodeficiency virus (HIV) 1 & 2 antibody/antigen	Negative		Negative
Hepatitis B surface antigen	Negative		Negative
Hepatitis C antibody	Negative		Negative
Syphilis screen			
Syphilis antibody	Positive		
*Treponema pallidum *hemagglutination assay (TPHA)	Positive		
Rapid plasma reagin (RPR)	Positive (1 in 64)		
*Treponema pallidum* antibody	Positive		
*Chlamydia trachomatis *nucleic acid amplification test (NAAT)	Negative		Negative
*Neisseria gonorrhoeae* NAAT	Negative		Negative
Cerebrospinal fluid (CSF) analysis			
Glucose	2.68	mmol/L	
Total protein	2.33	g/lL	0.1-0.4
White cell count	8 x 10^6^	cells/L	<5
Viral polymerase chain reaction (PCR)	Not detected		Negative
Culture	No growth		Negative
TPHA/RPR	TPHA-positive/RPR-positive (1 in 16)		

A sexually transmitted infection screen was also performed as part of the young-stroke workup and yielded positive syphilis serology (Table 1). This finding created diagnostic uncertainty regarding possible neurosyphilis, particularly because confirmation was delayed as lumbar puncture (LP) was initially considered relatively contraindicated due to ongoing dual antiplatelet therapy (DAPT). Consequently, a multidisciplinary team decision was made to initiate empirical treatment for neurosyphilis without delay.

Subsequent CSF analysis confirmed neurosyphilis, with positive *Treponema pallidum* hemagglutination assay (TPHA) and rapid plasma reagin (RPR) results. MRI and HR-VWI demonstrated features consistent with cerebral vasculitis, including multifocal vessel irregularity, beading, and concentric vessel wall enhancement (Figure 1), suggesting a vasculitic etiology rather than atherosclerosis. 

**Figure 1:**
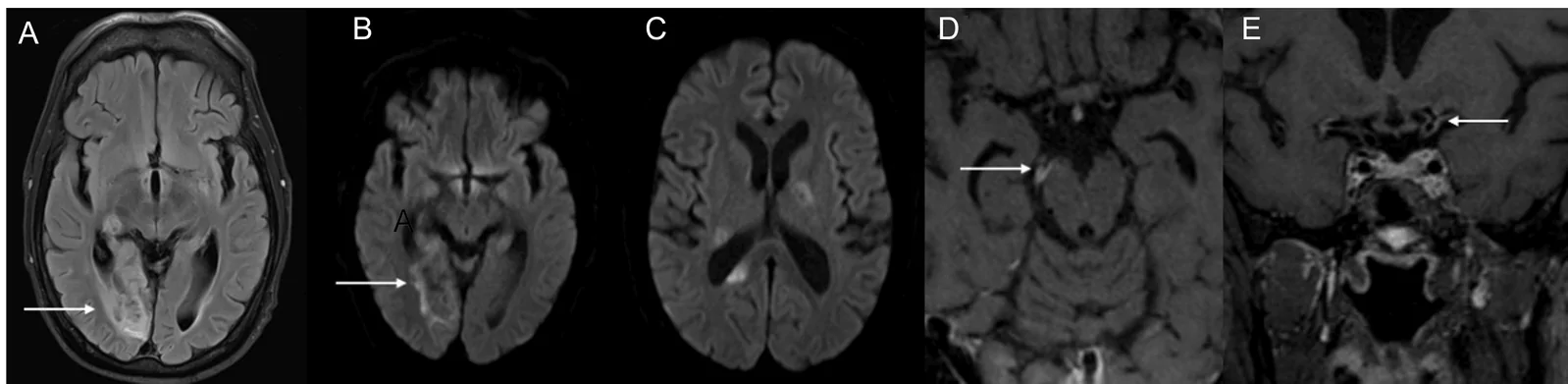
Brain MRI findings demonstrating multifocal acute-to-subacute infarcts and intracranial arterial wall enhancement in Case 1 (A) Axial fluid-attenuated inversion recovery (FLAIR) image demonstrating a subacute infarct in the right posterior cerebral artery (PCA) territory (arrow). (B) Axial diffusion-weighted imaging (DWI) b = 1000 image showing corresponding high signal intensity in the right PCA territory (arrow). (C) Axial DWI image demonstrating recent infarcts involving the genu of the left internal capsule, the right forceps major, and the right retrolenticular white matter. (D) Axial three-dimensional (3D) fat-saturated post-contrast SPACE sequence demonstrating concentric enhancement of the right PCA (arrow). (E) Axial 3D fat-saturated post-contrast SPACE sequence demonstrating similar concentric enhancement of the distal left internal carotid artery and the proximal left anterior cerebral artery and middle cerebral artery (arrow).

Treatment included a 14-day course of benzylpenicillin with corticosteroid cover to reduce the risk of a Jarisch-Herxheimer reaction, with follow-up guided by the serological response. Dual antiplatelet therapy (DAPT) was continued for three weeks following CSF analysis and then changed to lifelong clopidogrel monotherapy, in accordance with national secondary stroke prevention guidelines. Management involved a multidisciplinary team comprising specialists in stroke medicine, genitourinary medicine, neuroradiology, and microbiology, as well as rehabilitation therapists. The patient recovered sufficiently to mobilize independently without walking aids and perform all activities of daily living without assistance, with no residual neurological disability. 

Case 2  

A 48-year-old woman presented with significant dysarthria and left-sided hemiparesis involving the face, upper limb, and lower limb. Her medical history included a previous unexplained right thalamic and midbrain ischemic stroke six months earlier, hypertension, hypercholesterolemia, Raynaud’s phenomenon, anorexia, and chronic hyponatremia. Initial examination revealed left-sided facial droop and 4+/5 strength in the left elbow flexors, elbow extensors, and distal muscle groups. Coordination was impaired in both the left upper and lower limbs. No sensory deficits were identified. Three days after admission, the patient developed dysphagia, necessitating nasogastric tube insertion, along with worsening dysarthria.

Initial investigations included non-contrast CT of the head and CT angiography, which confirmed a right hemipontine perforator infarct, with basilar artery stenosis and occlusion of the left superior cerebellar artery and right posterior cerebral artery. Extensive investigations, including ambulatory electrocardiography (ECG), transthoracic and bubble echocardiography, antiphospholipid antibody screening, an autoimmune panel (including anti-Ro60, anti-Ro52, anti-La, anti-Sm, anti-Sm/RNP, anti-RNP68, anti-Scl-70, anti-Jo-1, anti-centromere, anti-chromatin, anti-ribosomal P, anti-myeloperoxidase, and anti-proteinase 3 antibodies), total homocysteine measurement, and blood-borne virus screening, were unremarkable (Table 2).

**Table 2:** Summary of laboratory findings in Case 2, with corresponding reference ranges

Investigation	Result	Units	Reference ranges
Plasma viscosity	1.85	mPa/S	1.50-1.72
Total cholesterol	3.28	mmol/L	<2.3
Hemoglobin A1c	42	mmol/mol	0-41
Total homocysteine	18	umol/L	0-18
Thyroid-stimulating hormone	2.3	mIU/L	0.2-4.3
Rheumatoid factor	<8	IU/mL	0-14
Autoimmune screen			
Antinuclear antibody (ANA) screen	Negative		Negative
Cardiolipin antibody (IgG)	<1.60	GPL U/ml	0-19.99
Beta-2 glycoprotein 1 antibody (IgG)	<1.40	U/ml	<20
Cryoglobulins	Not detected		Not detected
Complement C3	0.93	g/L	0.75-1.65
Complement C4	0.16	g/L	0.14-0.54
Immunoglobulins (Ig)			
IgG	19.8	g/L	6-16
IgA	1.9	g/L	0.8-4
IgM	0.98	g/L	0.36-2.89
Human immunodeficiency virus (HIV) 1 & 2 antibody/antigen	Negative		Negative
Hepatitis C antibody	Negative		Negative
TB interferon-gamma release assay	Negative		Negative
Herpes simplex virus type 1 (HSV-1) DNA (serum)	Negative		Negative
HSV-2 DNA (serum)	Negative		Negative
Adenovirus DNA (serum)	Negative		Negative
Varicella-zoster virus (VZV) DNA (serum)	Negative		Negative
Cerebrospinal fluid (CSF) analysis			
Glucose	3.89	mmol/L	
Total protein	0.53	g/L	
White cell count	54 x 10^6^	cells/L	
Culture	No growth		
HSV-1 DNA	Not detected		Negative
HSV-2 DNA	Positive		Positive
VZV DNA	Not detected		Negative
Adenovirus DNA	Not detected		Negative
Enterovirus RNA	Not detected		Negative
Cytomegalovirus(CMV) DNA	Not detected		Negative
Epstein-Barr virus (EBV) DNA	Not detected		Negative
Human herpesvirus 6 (HHV-6) DNA	Not detected		Negative

During the previous stroke admission, investigations revealed an elevated IgG kappa paraprotein level, suggestive of monoclonal gammopathy of undetermined significance (MGUS). However, normal platelet counts and negative paroxysmal nocturnal hemoglobinuria (PNH) and myeloproliferative neoplasm (MPN) screening results made this a less likely explanation for the recurrent thrombotic events. Alternative etiologies for the recurrent posterior circulation strokes were investigated using advanced imaging techniques. Bubble echocardiography showed no evidence of a right-to-left shunt, while magnetic resonance angiography (MRA) excluded vertebral artery dissection and atherosclerosis.

Subsequent HR-VWI demonstrated concentric enhancement of the intracranial arteries, supporting a diagnosis of cerebral vasculitis (Figure 2).  

**Figure 2:**
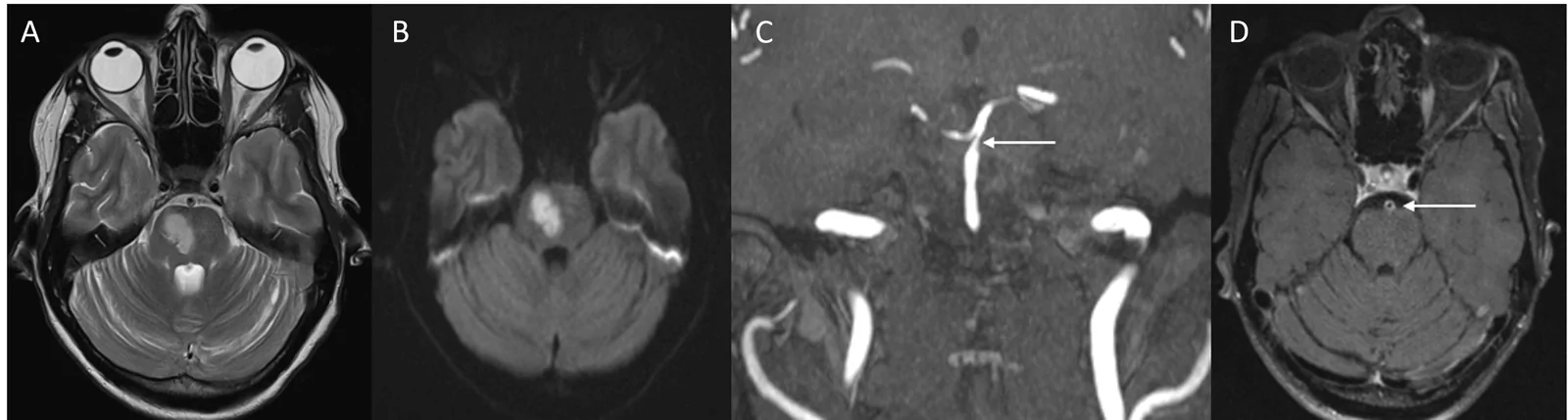
Brain MRI findings demonstrating acute pontine infarction with basilar artery stenosis and intracranial arterial wall enhancement in Case 2 (A) Axial T2-weighted image (T2WI) demonstrating right-sided pontine hyperintensity. (B) Axial diffusion-weighted imaging (DWI) b = 1000 image demonstrating corresponding high signal intensity in the right pons, consistent with an acute infarct. (C) Time-of-flight magnetic resonance angiography (TOF-MRA) demonstrating focal stenosis of the distal basilar artery (arrow). (D) Axial three-dimensional (3D) fat-saturated post-contrast SPACE sequence demonstrating concentric enhancement of the thickened basilar artery wall (arrow).

Following this, LP was performed. CSF analysis revealed elevated protein levels (hyperproteinorrachia) and a positive CSF polymerase chain reaction (PCR) result for HSV-2 (Table 2). In view of the otherwise unremarkable investigations summarized in Table 2, the positive CSF HSV-2 result was considered the most clinically significant finding, and treatment was initiated accordingly.

Treatment was initiated with intravenous aciclovir and intravenous methylprednisolone, which was subsequently tapered to oral corticosteroid therapy because of the suspected inflammatory vasculitic component. Serial CSF analyses were also arranged to monitor the clearance of the infection.

The final LP demonstrated persistent low-level HSV-2 DNA despite six weeks of intravenous aciclovir. Given the slow clearance of HSV-2 DNA from the CSF, a six-month course of oral valaciclovir was continued to prevent relapse, following consultation with a virologist. The patient's left hemiparesis improved markedly; however, she required percutaneous endoscopic gastrostomy (PEG) tube insertion because of persistent dysphagia. Management continued with a multidisciplinary approach focused on stroke prevention and rehabilitation.

## Discussion

Infection-associated cerebral vasculitis is a rare but important differential diagnosis in younger patients presenting with multifocal neurological deficits. Case 1 highlights the cerebrovascular complications of syphilis, which can affect the central nervous system at any stage of infection with *Treponema pallidum* and may remain clinically latent for years. Similarly, HSV-2 can remain latent and subsequently reactivate, rarely resulting in cerebrovascular complications, including vasculitis and ischemic stroke. These cases highlight the diagnostic challenges posed by neuroinfectious etiologies of ischemic stroke, as their clinical presentations are often non-specific and may resemble those of more common stroke mechanisms, which typically form the initial focus of clinical assessment. However, failure to consider an underlying infectious etiology may result in avoidable delays in treatment, potentially leading to significant neurological morbidity.

Although MRI and digital subtraction angiography (DSA) are useful techniques for identifying segmental arterial irregularities, the radiological findings of vasculitis may be mistaken for atherosclerosis and other non-inflammatory pathologies, as demonstrated in Case 1 [3]. MRI with HR-VWI uses signal suppression of luminal blood flow to directly visualize the vessel wall and associated pathology. In both cases, MRI with HR-VWI demonstrated a pattern of concentric vessel wall enhancement, consistent with a vasculitic process rather than atherosclerosis, which often demonstrates eccentric enhancement [4]. The radiological findings in both cases were instrumental in guiding further investigations.

CSF analysis was pivotal in both cases, yielding positive microbiological results that facilitated the identification of the causative pathogens and informed subsequent management.

In Case 1, LP was delayed because of the DAPT initiated for the suspected ischemic stroke with intracranial stenosis. Similarly, in Case 2, LP was delayed by six days because the patient was receiving clopidogrel monotherapy. This highlights a potential diagnostic challenge in the investigation of stroke syndromes, as antiplatelet therapy may complicate the timing of invasive diagnostic procedures when an infectious or inflammatory cause is suspected. A recent observational study involving 1,677 patients over a nine-year period concluded that adverse event rates following LP were similar among patients taking antiplatelet agents and those who were not [5]. These findings suggest that antiplatelet therapy may not be associated with a substantial increase in LP-related complications, although further evidence is required to establish procedural safety in this population. In the context of acute ischemic stroke, it is important to consider whether there are radiological features of raised intracranial pressure before performing LP to minimize the risk of complications, such as herniation. Conversely, earlier LP may facilitate timely diagnosis and management of the underlying etiology in selected patients, provided that relevant imaging findings and contraindications have been carefully assessed.

Although the Association of British Neurologists guidelines advise withholding clopidogrel for seven days before performing LP, it is important to note that most of the evidence underpinning this guidance is based on studies of spinal anesthesia rather than diagnostic LP specifically [6]. The guidelines also recommend that decisions regarding periprocedural antithrombotic management be made on an individual basis. This requires careful consideration of the clinical urgency of the diagnostic procedure and its implications for immediate management, alongside the competing risks of thrombosis associated with withholding antiplatelet agents and hemorrhage related to the invasive procedure. In challenging cases, advice should be sought from local specialist hematology services.

The management of infectious vasculitis varies according to the underlying etiology and may include pathogen-directed antimicrobial therapy alongside corticosteroid therapy to mitigate secondary inflammation. The precise mechanisms underlying HSV-associated vasculitis remain poorly understood [6], and the role of corticosteroids is therefore uncertain. Given the potential for immunosuppression to delay viral clearance, their use should be individualized according to the clinical context.

Untreated HSV-2 vasculopathy has been associated with progressive disease and may result in multifocal, recurrent stroke presentations [7], as demonstrated in Case 2, potentially leading to significant long-term disability. Therefore, timely and accurate diagnosis is essential to formulating a safe and effective management plan. Case 1 also highlights the benefits of early, targeted therapy for syphilis-related cerebrovascular complications, which resulted in significant functional and neurological improvement and ultimately facilitated safe discharge home. Long-term outcomes may also vary depending on the extent of intracranial involvement, particularly in the presence of multifocal lesions.

Furthermore, the multidisciplinary team, including neurology, microbiology, neuroradiology, and rehabilitation services, played an integral role in the diagnosis, management, and overall care of both patients.

## Conclusions

Neuroinfectious vasculitis should be considered in patients presenting with atypical, multifocal, or recurrent ischemic strokes, particularly when the clinical presentation and neuroimaging findings suggest an underlying inflammatory or systemic process. Although MRI with HR-VWI cannot establish the underlying etiology, it can identify features of vasculopathy and guide further investigations to determine the cause, including potential infectious and inflammatory etiologies. This is essential to facilitate the timely initiation of appropriate treatment. The decision to perform lumbar puncture in patients receiving antiplatelet therapy should be individualized, with specialist hematology input sought where appropriate. Early multidisciplinary collaboration is essential to establish the diagnosis and guide both acute and long-term management.
